# Single-cell sequencing technology in colorectal cancer: a new technology to disclose the tumor heterogeneity and target precise treatment

**DOI:** 10.3389/fimmu.2023.1175343

**Published:** 2023-05-15

**Authors:** Rongbo Wen, Leqi Zhou, Zhiying Peng, Hao Fan, Tianshuai Zhang, Hang Jia, Xianhua Gao, Liqiang Hao, Zheng Lou, Fuao Cao, Guanyu Yu, Wei Zhang

**Affiliations:** Department of Colorectal Surgery, Changhai Hospital, Naval Medical University, Shanghai, China

**Keywords:** colorectal cancer, single-cell sequencing, tumor heterogeneity, tumor microenvironment, precise treatment

## Abstract

Colorectal Cancer (CRC) is one of the most common gastrointestinal tumors, and its high tumor heterogeneity makes traditional sequencing methods incapable of obtaining information about the heterogeneity of individual cancer cells in CRC. Therefore, single-cell sequencing technology can be applied to better analyze the differences in genetic and protein information between cells, to obtain genomic sequence information of single cells, and to more thoroughly analyze the cellular characteristics and interactions in the CRC microenvironment. This will provide a more comprehensive understanding of colorectal cancer development and metastasis and indicate the treatment plan and prognosis. In this study, we review the application of single-cell sequencing to analyze the tumor microenvironment of CRC, explore the mechanisms involved in CRC metastasis and progression, and provide a reference for potential treatment options.

## 1 Introduction

Colorectal cancer (CRC) is one of the most common gastrointestinal tumors and is reported to have the third-highest morbidity and second-highest mortality worldwide (1). Additionally, approximately 50% of patients are diagnosed with metastatic CRC (mCRC) at the first visit, and the 5-year survival rate is no more than 50% (2). Tumor heterogeneity is a result of differences in genetic and molecular characteristics between individual cancer cells due to different degrees of cell differentiation in the same tumor tissue (3). The tumor microenvironment (TME) interacts with tumor heterogeneity to promote differentiation into different cell subtypes and metastatic spread, thus participating in the cancer development process (4, 5). CRC is highly heterogeneous; however, traditional sequencing methods target the entire tumor tissue, which can only reflect the total characteristics of the cell population and cannot obtain information on cell heterogeneity (6). Single-cell sequencing (SCS) sequences single cells at the genome or transcriptome level to obtain genomic, transcriptomic, or other information about individual cells, thereby revealing cell population differences and evolutionary relationships (7, 8). Therefore, studies conducted on individual cells can be more precisely localized to cell-to-cell interactions and are more conducive to detecting heterogeneity among individual cancer cells, thus exploring the complex heterogeneous mechanisms involved in the development of CRC, further clarifying diagnosis, improving prognostic analysis, and monitoring drug efficacy. Recently, SCS technology has been applicated in more and more studies and reported to help achieve considerable breakthrough in many other tumors including lung cancer, breast cancer and prostate cancer (9–11). Here, we review the studies of SCS in colorectal cancer and provide researchers with a reference for better understanding the tumor characteristics from multiple dimensions (**Table 1**) exploring its clinical application potential, and directing precise treatment.

**Table 1:** Overview of key findings in CRC SCS technology.

Type of Sample	Species	Cell Infiltration	Lineages represented	Key finding	Sequencing Technology	Reference
CRC tissues and nonmalignant colon tissues	Human	Tumor tissues: • SPP1 + macrophages • Th17 cells • Treg cells • CD8 + T cells • Myofibroblasts • IgG+ plasma cells Normal mucosa: • γδT cells • Matrix fibroblasts • CD4 + T cells • IgA+ plasma cells	Epithelia, immune	• An overall increase in myeloid cells and an overall decrease in B cells were observed in tumor tissue compared to normal tissue. • Human colon cancer cells have a multilineage differentiation process of normal colonic epithelial cells. • Myofibroblasts stimulate tumor growth through extensive tissue remodeling and support cancer stem cell survival through Wnt signaling. • The marked expansion of SPP1 + macrophages may play a central role in immunosuppression and tumor progression through osteopontin, and patients with high levels of SPP 1 + expression have a poor prognosis. • T cell subtypes are evenly distributed in normal and tumor tissues: Th17 and Treg cells are mainly distributed in tumor tissues, and γδT cells are abundant in normal mucosa.	10X Chromium	(12)
Paired cancer and normal organoids from microsatellite-stable EOCRC patients	Human			• The observation of molecular phenotypic diversity, including PTPRK-RSPO3 fusions. • Discovery of the similarity between RSPO fusion-like organs and normal-like organs, with high BMP2 and low PTK7 expression.	WES, WGS, 10X Chromium	(13)
Tumor PDOs	Human			• Discovery of the heterogeneity of copy number alterations in tumor PDOs. • Chromosomal instability and karyotype evolution persist in human colon cancer carcinoids.	scKaryo-seq	(14)
Fresh normal colon and colorectal cancer tissues	Human	Definition of six transcriptome-based states of CRC cells: • Stem/TA-like • Goblet cell-like • TC1-4 Be found in colorectal polyp and cancer cell: • Stem/TA-like TC1-4	Epithelia, immune	• Definiton of patient-overarching colorectal cancer cell clusters characterized by differential activities of oncogenic signaling pathways such as mitogen-activated protein kinase and oncogenic traits such as replication stress. • CRC cell development trajectories follow the MAPK pathway in tumor organoids. • The targeting of EGFR-BRAF-MEK in tumor organoids depends on acquired KRAS/BRAF mutations and induced cellular plasticity rather than the default developmental trajectory that affects signal transduction and gene expression.	10X Chromium	(15)
CRC tissues and adjacent normal intestinal crypts	Human			• CRC cells carry several times more somatic mutations than normal colorectal cells, which is more likely acquired during the final dominant clonal expansion of the cancer. • Genetic diversification of each cancer is accompanied by pervasive, stable and inherited differences in the biological states of individual cancer cells.	single-cell genome sequencing, WGS	(16)
Lung cancer, CRC, ovary cancer and breast cancer tissues	Human	• B cells • Treg cells • CD8+ T cells CD4+ T cells	Endothelia, immune	• T cells are the most common cell type found in tumor tissue. • Tissue specificity of endothelial cells is restricted to normal tissue. • Fibroblasts are a cell type shared by a variety of tissues, showing the highest cancer type specificity. • Colon-specific subsets mainly exist in normal tissue. • Dendritic cells and T cells present low -tissue-specific. • B cells, except plasma cells in mucosa-rich normal colon, all other tumors are rich in B cells. • In myeloid cells, other myeloid cell subsets exist in all cancer types, except for the resident alveolar macrophages.	10X Chromium CITE-seq	(17)
CRC tissues, Liver metastasis tissues and adjacent tissues	Human Mouse	CRLM: • CD8+ T cells • CD4+ T cells • NK cells • B cells Liver Metastasis: • SPP1+ macrophages • MRC1+ macrophages • CCL18+ macrophages	Immune	• Further developed scMetabolism, a computational pipeline to quantify single-cell metabolism, and observed that these macrophages have enhanced metabolic activity. • Provided a single-cell and spatial map of colorectal liver metastases and identified highly metabolically activated MRC 1 + CCL 18 + m2-like macrophages at the metastatic site. • Efficient neoadjuvant chemotherapy can slow the metabolic activation, increasing the possibility of targeting metabolic pathways in metastasis.	10X Chromium	(18)
CRC tissues and adjacent normal tissues	Human	• Tumor tissues: • CTLA4+ Tregs • CTLA4- Tregs • Th 1/Th 17 cells with high CXCL13 expression • Macrophages Adjacent normal tissues: • Naive T cells (CD8-TCF 7 and CD4-CCR 7) • Tex cells(CD8- HAVCR2)	Immune	• CXCL13 + T cells may perform similar functions in microsatellite unstable (MSI) tumors and are associated with a high response rate to checkpoint blockade. • Discovery of reduction of antigen presentation and anti-tumor immunity of CD40 + and CD27 + cells in tumors. • Communication between non-immune cells and immune cells expanded significantly in the tumor.	10X Chromium	(19)
CRC tissues and liver metastasis	Human	• B cells(early tumor) • Plasma cells(advanced tumor): IgA+IGLC2+ plasma cells	Immune	• B cells in early CRC tumors are predominantly pre-B-like cells with tumor suppression capacity, whereas B cells in advanced CRC tumors develop as plasma cells. • The interaction between CCL 8 + cycling B cells and CCR 5 + T cells may play an antitumor role in advanced CRC. • T-cell anti-tumor responses are activated in CRC tumors, and in the tumor microenvironment, T-cell responses are attenuated by myeloid cells.	Smart-seq2 DNBelab C4	(20)
CRC tissues and adjacent normal tissues	Human	• CD8+ Tem cells • Tex cells • Effector T cells • Th17 cells • Th1-like cells with high expression of CXCL13 • Intraepithelial lymphocytes(CD160+)	Immune, endothelia	• Together, the tumor microenvironment and TCR affected the transformation of tumor-infiltrating CD8 effector memory T cells to exhausted T cells and effector T cells. • Among CRC patients, MSI/dMMR patients showed a significantly better therapeutic response to immune checkpoint inhibitors than did MSS patients. • Intraepithelial lymphocytes and Th 17 cells were more enriched in CRC patients than in liver and lung cancer.	Smart-seq2	(21)
CRC tissues, liver metastasis tissues, blood and adjacent normal tissues	Human	• CD8+T cells a. IEL b. Tex c. MAIT • CD4+T cells a. Th17 b. Th1-like c. IL10+ Treg d. CTLA4+ Treg • Macrophages a. FCN1+ RTM b. C1QC+ TAM c. NLRP3+ RTM d. PLTP+ RTM e. CXCL12+ RTM f. MKI67+ TAM g. SPP1+ TAM • DCs a. LAMP3+ cDC b. CD1C+ cDC2 c. DC3 d. FCN1+ cDC2 e. TIMP1+ cDC2	Immune	• The CD8+ Texs are malignancy-related and TCR-dependent. • Primary CRC tumors with Liv.Mets may exhibit a stronger immunosuppressive niche compared to non-metastatic CRC tumors. • SPP1+ is mainly found in Liv.Mets as well as the potent phagocytosis of C1QC+ and may play an important role in tumor metastasis. • Proinflammatory DC3 is phenotypically formed by cancer cells in the CRLM.	Smart-seq2	(22)
CRC tissues	Human Mouse	• Treg cells • CD8+ T cells	Immune	• Intratumoral Tregs are characterized by low activity of the MondoA-thiokycin interacting protein (TXNIP) axis and increased glucose uptake. • Inhibition of the MondoA-TXNIP axis promotes glucose uptake and glycolysis, inducing Th 17-like Tregs with high glycolysis, thereby promoting Th 17 inflammation, promoting interleukin 17A-induced CD8+ T cell exhaustion, and driving colorectal cancer. • IL-17A blockers can be coordinated with PD-1 inhibitors in treating AOM-DSS-induced colorectal cancer.	Smart-seq2	(23)
CRC tissues and adjacent normal tissues	Human Mouse	• C1QC+ TAMs • SPP1+ TAMs • Tem cells • Trm cells • DCs • Bhlhe40+ TH1-like cells • CD8+ Tm cells	Immune, mesenchyme	• C1QC+ TAM interacts with a variety of T cells and plays the function of cell phagocytosis and antigen presentation. • SPP1+ TAM mainly interacts with fibroblasts to play the function of promoting angiogenesis and promoting tumor metastasis. • The anti-CSF1R blocking antibody affects the proliferation of macrophages during the cell cycle, specifically deleting a percentage of macrophages with C1QC+ TAM features, but not on macrophages with SPP1+ TAM features. • α CD40 agonists can exert their immunotherapeutic effects by activating DC cells, promoting Bhlhe40 + Th 1 cells, enhancing the migration ability of Tem cells between lymph nodes and tumors and the conversion ability between Tem cells and Trm cell groups.	Smart-seq2	(24)
CRC tissues	Human Mouse	• ILC1s • ILC2s(ILC2-A, ILC2-B, ILC2-C) • ILC3s • ILCregs	Immune	• ILC1s express inhibitory receptors and undergo inhibitory functional turnover in late CRC. • ILC2-C can promote tumor progression. HS3ST1 and PD1 are highly expressed in ILC2 in advanced CRC tumors, and lack of HS3ST1 or PD1 in ILC2s inhibited tumor growth. • ILC3s transdifferentiate into ILCregs during CRC progression, and ILCregs promotes tumor growth.	10X Chromium	(25)
CRC tissues	Human	• CSCs	Epithelia, Stem cells	• Rare CSCs in CRCs exist in a dormant state and possess high stemness and high WNT, TGF-β and YAP/HIPPO signaling and are able to maintain short telomeres without cell proliferation.	10X Chromium Smart-seq2	(26)

## 2 Application of SCS for in-depth analysis of CRC TME

The TME is a complex integrated system composed of tumor cells, various cytokines, chemokines, and multiple stromal cells, including immune inflammatory cells, cancer-associated fibroblasts, and endothelial cells. These can be divided into immune microenvironments, which are dominated by immune cells, and non-immune microenvironments, which are dominated by fibroblasts (27). Tumor cells are closely related to TME. Tumor cells can influence its microenvironment by releasing cell signaling molecules to promote tumor angiogenesis and induce immune tolerance, while immune cells in the microenvironment can influence tumor cells growth and development. Several studies have shown that changes of TME are closely related to cancer development, invasion, and metastasis, and immunotherapy efficacy can be predicted by distinguishing different TME subtypes (28–30). Therefore, the application of SCS technology for in-depth analysis of the CRC TME is a hot research area, and many research results have laid the foundation for future molecular typing and treatment of CRC.

### 2.1 Tumor cells

CRC is characterized by genomic instability, epigenetic abnormalities, and abnormal gene expression (31). This has led to high tumor heterogeneity in patients with CRC. Tumor heterogeneity in CRC can be divided into inter- and intra-tumor heterogeneity. CRC inter-tumor heterogeneity may originate from human embryonic development, where different embryonic layers develop as proximal and distal colon, and hypermutated tumors with microsatellite instability (MSI) are often located in the proximal colon, while tumors in the distal colon or rectum usually exhibit microsatellite stability (MSS) and chromosomal instability (CIN) (32). CRC intra-tumor heterogeneity (ITH) may also occur due to differences in cancer cells and TME within the tumor, further complicating the development of new therapeutic strategies and biomarker identification (33). ITH is detectable within a single tumor in which cancer cell subpopulations with different genome features coexist in a patient in different tumor areas or may differ over time (34–36). Exploring the ITH within single tumor is the advantage of SCS technology.

CRC cells are similar to normal cells to some extent but exhibit individualized phenotypic diversity at the same time. Through analyzing the single-cell RNA sequencing (scRNA-seq) data of metastatic CRC patients, Lee HO et al. found that the differentiation trajectory of CRC cells was similar to that of normal epithelial cells, with aggregation of tumor cells with normal stem cell-like/transporter-amplified cell populations. This suggests that tumor epithelial cells have regenerative and proliferative potential, while clustering analysis showed that tumor epithelial cells have highly variable transcriptional status and individual variability (12). Another study also confirmed the similarity between CRC-like organs and normal-like organs at the patient-derived tumor organoids (tumor PDOs) level, but with molecular phenotypic diversity. scRNA-seq analysis of paired cancer and normal tissue organoid models from patients with MSS sporadic early-onset CRC (EOCRC) resulted in the observation of significant molecular phenotypic diversity, including PTPRK-RSPO3 fusions, and RSPO fusion organoids were similar to normal colon organoids with high BMP2 and low PTK7 expression, which confirms the similarity between RSPO fusion organs and normal organs (13). In addition, a study confirmed the heterogeneity of copy number alterations in tumor PDO by single-cell karyotype sequencing and showed that monoclonal lines evolved new karyotypes over time *in vitro*, suggesting that chromosomal instability and karyotype evolution persist in human colon cancer carcinoids (14). In another study using tumor PDOs and biopsy tumors, it was demonstrated that karyotypic alterations of varying complexities are prevalent and can occur within several cell generations (37). Both studies corroborate the existence of complex karyotypic alterations and evolution in CRC, resulting in high intratumoral heterogeneity.

Meanwhile, RNA metabolic markers and RNA velocity assessments revealed CRC cell development trajectories along the mitogen-activated protein kinase (MAPK) pathway in tumor PDOs, whereas normal colon organoid cells develop along a hierarchy of WNT activity, highlighting that cancer cell development is a driver of non-genetic cancer cell heterogeneity and that changes in trajectories affect the effectiveness of targeted therapies (15). Roerink et al. concluded that CRC cells exhibit extensive mutational diversity and carry several times more somatic mutations than normal colorectal cells. Most mutations are acquired during the final dominant clonal expansion of cancer cells and are caused by mutations that are absent in normal colorectal cells. Specific somatic mutations can result in different responses to chemotherapy and targeted therapy (16). This suggests that CRC cell development, clonal expansion, and mutations continue to drive intratumor heterogeneity in CRC, making chemotherapy and targeted therapy difficult and promoting drug resistance in patients.

To deeply explore CRC intra-tumor heterogeneity, a study performed optimized single-cell multi-omics sequencing, including DNA, DNA methylation, and transcriptome sequencing, on CRC patients with CRC. This showed that DNA methylation levels in CRC cells were lower than those in normal epithelial cells adjacent to the cancer cells, with different methylation levels varying from different spectra in the same tumor tissue, suggesting that methylation heterogeneity mainly results from differences in DNA methylation between different subclones within the same patient’s tumor. This study also elucidated the demethylation characteristics of CRC, where the degree of demethylation was consistent within each subtype but varied across subtypes. Interestingly, long interspersed nuclear element 1 (LINE-1, L1) shows stronger demethylation than L2 in cancer cells, in contrast to embryonic development, suggesting that abnormal demethylation processes may arise in the L1 and heterochromatin regions during tumorigenesis and progression, breaking the normal developmental pattern (38).

### 2.2 Immune cells

#### 2.2.1 T cell

Various immune cells in the TME interact with tumor cells and mediate immune tolerance to tumors, affecting tumor progression and metastasis, and thus immunotherapy efficacy. T cells, one of the major cellular components involved in the body’s immune response and the most common cell type in tumor tissue (17), can kill tumor cells. In order to escape the pursuit of T cells, tumor cells could produce some inhibitory signals on their own surface, and inhibit the immune function of T cells through immune checkpoint (39). Ever since Allison et al. discovered the immunosuppressive effects of CTLA4 on T cells in 1996, immunotherapy drugs, notably immune checkpoint inhibitors (ICIs), have taken off and become a lifesaving drug for tumor patients (40). Various ICIs have been applicated in cancer immunotherapy, including PD1/PDL1 and CTLA4 (41, 42).

However, the efficacy of immunotherapy in patients with CRC is not as good today. Thus, an increasing number of studies have been conducted to analyze the TME in depth using SCS technology.

Several studies have mapped the global cellular landscape in CRC, with an overall increase in myeloid cells and an overall decrease in B-cell numbers observed in tumor tissues compared to normal tissues, suggesting a redirected immune response. This indicates that the immune response undergoes dynamic changes during cancer development and that the transcriptional profile of cancer cells is similar to that of normal human differentiation, with genetic alterations creating an immunosuppressive microenvironment directed by regulatory T cells (Tregs), myofibroblasts, and myeloid cells (12, 18–20). Meanwhile, T cell subtypes are unevenly distributed in normal and tumor tissues, Th17 and Treg cells are mainly distributed in tumor tissues, while γδT cells are more abundant in normal mucosa. The TME and T-cell receptors (TCR) affect the transformation of tumor-infiltrating CD8+ effector memory T cells (Tems) into exhausted T cells (Texs) and effective T cells (Teffs), indicating the transformation of the organism from mucosal immunity to inhibiting cellular immunity (12, 21).

A study analyzing CD45+ cells from multiple matched tissues of patients with untreated primary hepatocellular carcinoma (HCC), CRC, and CRC liver metastases (CLM) found that Texs and activated Tregs originate from primary CRC tumors with a malignancy-related phenotype and are TCR-dependent. There is a high degree of TCR sharing between Tems and Texs. Natural killer (NK) cells and mucosa-associated T cells are mainly derived from the liver tissue at metastatic foci, and their phenotypes are associated with the liver TME (22). Another study similarly found decreased B-cell antigen presentation as well as tumor-specific Tregs and their two subtypes, proliferative exhausted T cells and a predominance of naive T cells (CD8-TCF7 and CD4-CCR7) in adjacent tissues and exhausted T cells in tumors (CD8-HAVCR2). Th1/Th17 cells with high CXCL13 expression are preferentially enriched in patients with a high tumor mutational burden (TMB) and respond well to immunotherapy (19), suggesting that CXCL13+ T cells may perform functions similar to those of MSI tumors and are associated with a high response rate to checkpoint blockade. The results of these two studies are consistent, suggesting that the exhausted T cell phenotype is associated with malignancy and that CXCL13+ T cells may be associated with immunotherapy. Moreover, CXCL13 expression accurately identifies both tumor-specific T cell clones that are terminally differentiated and highly exhausted and tumor-specific T cell precursor cell clones that are abundantly present in responding tumors after immune checkpoint blockade (ICB) treatment, demonstrating that tumor-specific CXCL13+CD8+ T cells play a key role in the treatment process and that the degree of infiltration before treatment can predict ICB efficacy (43).

It was also found that in tissue samples from CRC patients, impaired T cell proliferation and activation were associated with ZFP91, which disrupts the metabolic pathways and antitumor activity of tumor-infiltrating T cells, suggesting that targeting ZFP91 may improve the effectiveness of tumor immunotherapy (44). The transcription factor TCF-1 is critical for Treg development and function and primarily inhibits the transcription of genes that co-bind with FOXP3. Deficiency of TCF-1 could activate Treg cells and make Th17 cells acquire the intestinal homing characteristics, leading to more dangerous and dramatic CRC, and the specific TCF-1 expression of Tregs regulates inflammation and CD8+ T cell toxicity and may determine the prognosis of CRC (45). Similarly, the glucose-responsive transcription factor MondoA is highly expressed in Tregs, and inhibition of the MondoA-TXNIP axis promotes glucose uptake and glycolysis, inducing highly glycolytic Th17-like Tregs, which promotes Th17 inflammation and CRC development (23).

#### 2.2.2 Cancer-associated fibroblasts

In addition to T cells, the most common cells in the TME, cancer-associated fibroblasts (CAF), and tumor-associated macrophages (TAMs), are also being explored and analyzed with the development of SCS technology.

Somatic copy number alterations (SCNAs) are prevalent in the TME and immune cells, fibroblasts, and endothelial cells in the normal tissues of each individual, and the proportion of fibroblasts with genomic copy number variants is much higher in tumor tissues than in adjacent issues, so that it can predict the prognosis of CRC by screening the differentially expressed genes of CAFs in tumor tissues. Five genes (BGN, RCN3, TAGLN, MYL9, and TPM2) have been identified as specific CAFs biomarkers of poor prognosis in CRC (46). Fibroblasts are a cell type common to multiple tissues and exhibit the highest cancer-type specificity (17). In addition, myofibroblasts have been shown to stimulate tumor growth through extensive tissue remodeling and support cancer stem cell survival through WNT signaling (12), indicating that fibroblasts also play a role in promoting tumor growth, and that genetic alterations in fibroblasts could incur CRC *via* paracrine signaling in epithelial cells (47).

#### 2.2.3 Tumor-associated macrophages

TAMs are infiltrating macrophages in tumor tissue, mainly derived from monocyte differentiation. TAMs can interact with tumor cells through exosomes or secrete multiple cytokines to promote tumor cell proliferation, invasion, migration, and angiogenesis.

TAMs recruit Tregs through chemokine CCL2 secretion, which inhibits the antitumor immune response of T cells and interferes with immune cell interactions, thus leading to an immunosuppressive microenvironment in CRC (48). TAMs in CRC can be divided into two cell groups, SPP1+ TAM and C1QC+ TAM. C1QC+ TAM interacts with various T cells and performs cytophagic and antigen-presenting functions, whereas SPP1+ TAM interacts mainly with fibroblasts and performs pro-angiogenic and tumor-promoting functions (24). Single-cell analysis of CLM samples showed that a subpopulation of dendritic cells (DC3s) and SPP1+ macrophages is associated with malignancy and plays a critical role in liver metastasis (22). Additionally, a study detected numerous immunosuppressive cells in CRC liver metastatic tumors, with a dramatic increase in SPP1+ macrophages and MRC1+ CCL18+ macrophages and an enrichment of neutrophils as potential participants in liver metastases (18). A previous study also showed that significant expansion of SPP1+ macrophages may play a central role in immunosuppression and tumor progression through bone bridge proteins and that CRC patients with high SPP1+ expression levels have a poorer prognosis (12). These studies confirm the role of SPP1+ macrophages in promoting CRC progression and the potential suppressive TME in liver metastasis. In addition, one study showed that the density of TAMs was not associated with survival in patients with CLM, but the area and circumference of TAMs were significantly higher in CLMs, while larger morphologies of TAMs were mostly observed in patients with a poorer prognosis (49), which demonstrated the strong prognostic significance of the morphological representation of TAM.

#### 2.2.4 Intrinsic lymph-like cell

Intrinsic lymphocytes (ILCs) are located on the mucosal surface and include NK cells; helper classes ILC1s, ILC2s, and ILC3s; and lymphoid tissue-inducing (LTi) cells that enhance the immune response, maintain mucosal integrity, and sustain tissue homeostasis (25, 50).

One study analyzed tumor-infiltrating ILCs during CRC progression using scRNA-seq and classified them into six clusters. ILC1 expressed inhibitory receptors and underwent an inhibitory functional transformation in advanced CRC, and ILC2 was divided into three subgroups (ILC2-A, -B, and -C), of which the ILC2-C subgroup promoted tumor progression. HS3ST1 and PD1 are highly expressed in ILC2 of advanced CRC In addition, ILC3 transdifferentiates into ILCregs during CRC progression and promotes tumor growth. Notably, the TGF-β signaling pathway initiated the conversion of ILC3 to ILCregs. Therefore, this study suggests that interfering with ILC conversion may be a potential strategy for CRC immunotherapy (25). Moreover, another study reported single-cell characteristics of blood and intestinal helper ILC subtypes in healthy conditions and CRC, where the healthy intestine contained ILC1s, ILC3s, and ILC3/NKs, but not ILC2s, while additional tumor-specific ILC1 and ILC2 subtypes were identified in CRC patients. SLAMF1 (signaling lymphocyte-activating molecule family member 1, CD150) was selectively expressed on tumor-specific ILCs, and higher levels of SLAMF1+ ILCs were observed in the blood of patients with CRC. The survival rate of patients with CRC in the high SLAMF1 group was significantly higher than that in the low SLAMF1 group, indicating that SLAMF1 is an antitumor biomarker of CRC (50).

## 3 Application of SCS to explore mechanisms affecting CRC metastasis & progression

### 3.1 CRC liver metastasis

CLM is the leading cause of death from CRC and a major factor in reducing the survival time of CRC patients (51). Besides the rapid metastatic spread of cancer cells, TME with liver metastasis exhibits a highly immunosuppressive phenotype (52). A previous study observed a dramatic increase in SPP1+ and MRC1+ CCL18+ macrophages in metastatic tumors, which corroborated the potentially suppressive TME in liver metastases. Meanwhile, TAM may be suppressed in metastatic tumors, liver metastatic cells may preferentially reprogram macrophages and induce their specific functional states, and metastatic tumor cells in liver metastases preferentially express the ligand CD47 and thus may recruit or activate MRC1+ CCL18+ macrophages through the corresponding receptor SIRPA, suggesting that specific macrophage subpopulations may play a fundamental role in the formation of premetastatic niche in CLM (18).

In addition, studies have revealed rare mutations in metastatic tumors and defined two separate cell populations by analyzing single-cell sequencing data from primary and metastatic foci in patients with CRC and liver metastases, suggesting different evolutionary trajectories between primary and metastatic tumor cells. Meanwhile, extensive WES data reflect different mutant allele frequencies between primary and metastatic foci. TP53, APC, and SNVs in SMAD4 show an increase in variant allele frequency (VAF) in metastatic samples (53).

### 3.2 Cancer stem cells

Cancer stem cells (CSCs) are thought to proliferate extensively and drive tumor growth, indicating that malignant cell populations in tumors are generated by CSCs (54). A previous study found that each tumor gland was derived from a stem cell by genomic analysis of 349 individual tumor glands, and they found that after initial transformation, CRC tumors grew primarily as a single expansion comprising many intermixed subclones, and it was then proposed that most of the mutations driving tumor growth occurred during early tumor expansion and led to clonal diversity and intra-tumor heterogeneity (55).

Currently, it has been suggested that CSCs may contribute to tumor progression and drug resistance. By single-cell sequencing of telomerase and transcriptome in 8 primary foci of untreated CRC, it was shown that CSCs can be remodeled into cancer epithelial cells and both of them retain the important signaling pathway such as WNT, TGF-β, and HIPPO/YAP. In addition, proliferating tumor epithelial cells were found to be derived from resting CSCs, which are related to the recurrence and metastasis of tumors, and resting CSCs may develop drug resistance through mutations (26).

### 3.3 CRC genomic/chromosomal mutations

Continued high-frequency chromosomal instability (CIN) has a dramatic impact on tumor evolution and treatment response. A previous study showed that CIN is prevalent in CRC, and single-cell karyotyping sequencing confirmed the heterogeneity of copy number alterations in tumor PDOs and showed that monoclonal lines evolved new karyotypes over time *in vitro (* 14). Abnormal DNA methylation at the chromosomal level has also been found in CRC cells, where six chromosomes (chromosomes 4, 5, 8, 13, 18, and X) tend to undergo intense DNA demethylation, with three hypomethylated chromosomes (chromosomes 8, 13, and 18) (46), which confirms that CRC is characterized by genomic instability.

In addition, it has been found that CRC cells usually develop along the mitogen-activated protein kinase (MAPK) pathway, while MAPK activity will drive the cellular trajectory of cancer cells, and the targeting of EGFR-BRAF-MEK in tumor-like organs depends on acquired KRAS/BRAF mutations and induced cellular plasticity, which affects signal transduction and gene expression (15). Furthermore, a study showed a significantly increased somatic mutation rate in CRC cells compared to normal colorectal cells; the presence of driver mutations such as BRAF (V600E), PIK3CA (E81K), and ACVR2A (protein truncated small indel); as well as MLH1 methylation and genetic diversification in each cancer, accompanied by generalized, stable and genetic differences (16).

## 4 Application of SCS to explore and improve CRC treatment

### 4.1 Immunotherapy targets

Current immunotherapies for metastatic CRC are effective only in tumors with high microsatellite instability or mismatch repair defects. As tumor cells can determine their immune microenvironment and often form an immunosuppressive microenvironment (12), current immune checkpoint inhibitors (ICIs) are not effective against tumors with proficient mismatch repair (pMMR), MSS, or low-frequency microsatellite instability (MSI-L) (called pMMR-MSI-L tumors) (56). Therefore, finding new immunotherapy targets or improving current immunotherapy to expand the range of CRC immunotherapies has become an area of research interest.

A study found that T-cell antigen receptor-dependent cytoplasmic translocation of ZFP91 promotes the assembly of the PP2A complex, thereby limiting mTORC1-mediated metabolic reprogramming, suggesting that ZFP91 interferes with the metabolic and functional state of T cells in the TME, suggesting that targeting ZFP91 may improve the efficacy of CRC immunotherapy (44).

In addition, a study indicated that anti-CSF1R treatment preferentially depletes macrophages with inflammatory features but avoids macrophage populations expressing pro-angiogenic/tumorigenic genes in mice and humans. Treatment with CD40 agonist antibodies preferentially activates conventional dendritic cell (cDC) populations with increased Bhlhe40+ Th1-like cells and CD8+ memory T cells and identified key cellular interactions that regulate tumor immunity and mechanisms for myeloid-targeted immunotherapy currently in clinical trials (24).

### 4.2 Causes of chemotherapy sensitivity

Currently, the primary treatment for CRC is surgical resection combined with radiotherapy, chemotherapy, and targeted therapy. However, some patients undergoing chemotherapy may develop drug resistance, resulting in reduced efficacy.

One study developed a scMetabolism system to provide a single-cell and spatial atlas of colorectal liver metastases and identified highly metabolically activated MRC1+ CCL18+ M2-like macrophages at metastatic sites. Efficient neoadjuvant chemotherapy can slow metabolic activation and increase the possibility of targeting metabolic pathways in metastases (18).

In addition, another study confirmed the strong response of the CMS2 epithelial/typical group to EGFR and HER2 inhibitors by translating consensus molecular typing (CMS) preclinical models of developing cancer cells adapted to CMS classifiers combined with high-throughput drug sensitivity screening and revealed that cells with CMS1 microsatellite instability/immunity and CMS4 mesenchymal phenotypes were strongly sensitive to HSP90 inhibitors. A combination of 5-fluorouracil and HSP90 inhibitor has the potential to relieve drug resistance and improve treatment efficacy in a CMS4 patient-derived xenograft (PDX) model (57).

## 5 Summary

Tumor heterogeneity is widespread in CRC patients, and cluster analysis shows that tumor epithelial cells are individualized for each patient and are highly mutated Various immune cells and inflammatory chemokines in the TME interact and influence each other to promote tumor progression, thus affecting tumor recurrence and treatment response and adversely affecting the prognosis of CRC patients. While the impact of the TME on CRC can be fully investigated by obtaining information on cancer cell characteristics through SCS, it is also possible to identify relevant predictive markers for CRC prognosis and potential immunotherapeutic targets through SCS, thus improving patient prognosis and therapeutic effects. In addition, the use of SCS technology for drug development and for addressing the problem of chemotherapy resistance in some patients is now emerging (31). It is expected to be applied in precision medicine to develop and personalize cancer medical treatments, providing a more accurate diagnosis and the best-individualized treatment plan for cancer patients.

However, SCS technology still has some limitations in current platform. First, SCS technology requires high levels of sample preparation and sample quality, including cell quantity and activity, which increased the combined cost. Second, although the number of cells that can be detected from SCS has increased from 10~100 to tens of thousands with the development of technology, the tedious process still results in the loss of some cell populations, which can bias the results. In addition, SCS technology lacks of spatial information. Thus, a combination of multiple sequencing methods including bulk sequencing, spatial transcriptomics and SCS technology could be a solution and future direction of development.

## Author contributions

WZ, GY and FC designed the research. RW, LZ and ZP made substantial contributions to acquisition, analysis and interpretation of data, and wrote the manuscript. HF, TZ, HJ, XG, ZL and LH coordinated and were involved in acquisition, interpretation of the data. WZ, GY and FC revised it critically for important intellectual content and gave final approval of the version to be published.
